# Acknowledgment to Reviewers of *Geriatrics* in 2020

**DOI:** 10.3390/geriatrics6010009

**Published:** 2021-01-26

**Authors:** Geriatrics Editorial Office

**Affiliations:** MDPI AG, St. Alban-Anlage 66, 4052 Basel, Switzerland

Peer review is the driving force of journal development, and reviewers are gatekeepers who ensure that *Geriatrics* maintains its standards for the high quality of its published papers. Thanks to the cooperation of our reviewers, in 2020, the median time to first decision was 18 days and the median time to publication was 47 days. The editors would like to express their sincere gratitude to the following reviewers for their precious time and dedication, regardless of whether the papers were finally published:
Abeare, Christopher A.Maeda, KeisukeAdamis, DimitriosMagnavita, NicolaAhmad, KhurshidMagriplis, EmmanuelaAhn, NayoungMaixent, Jean MichelAlalwan, TariqMandini, SimonaAntipova, VeronicaMarković Denić, LjiljanaAspinall, Peter J.Marottoli, Richard A.Athanasia, PrintzaMars, MauriceAyán, CarlosMarshall, SkyeAzzolino, DomenicoMaruya, KoheiBaeyens, Jean-PierreMasala, CarlaBagiu, Iulia-CristinaMazzola, PaoloBagyinszky, EvaMcCarthy, GeraldineBaker, Brent A.Mędrzycka Dąbrowska, WiolettaBalzarro, MatteoMentes, Janet C.Barberis, FabrizioMento, CarmelaBaughman, Amy WisteriaMiddleton, MarkBaum, JamieMilazzo, MarioBehrens, LizaMolero, PatricioBerton, FedericoMontemurro, NicolaBessi, ValentinaMonzani, FabioBevc, SebastjanMorey, Miriam C.Bevilacqua, RobertaMugueta-Aguinaga, IranzuBirt, LindaMuhammad, AsgharBoaden, ElizabethMura, GioiaBoccardi, VirginiaMurata, ChiyoeBravo-Aguilar, MaríaNguyen, Tu N.Bruins Slot, KarstenNiklasson, JohanBrunetti, EnricoNjemini, RoseBuckley, RichardNolan, ClodaghBujnowska-Fedak, Maria MagdalenaO’Brien, MaritaBullo, ValentinaO’Caoimh, RónánBurgos, RosaO’Donovan, SineadByeon, HaewonOgawa, AsaoCallegari, CamillaOlczyk, PawełCapelli, CarloOlmedillas, HugoCaron, RosemaryOno, YukoCastellani, DanielePachana, NancyCatic, Angela GeorgiaPacheco-da-Costa, SorayaCauli, OmarPaire-Ficout, LaurenceCermakova, PavlaPanagiotopoulos, Elias C.Chandler, Melanie J.Parrish, RichardChapman-Novakofski, KarenPartridge, JudithChen, Yi-MingPassantino, AndreaChien, Jung-YienPatel, Harnish P.Christ, MichaelPellegrino, GerardoChui, KevinPeng, XianChurch, Frank C.Pérez Navío, EufrasioClark, CollinPérez-Ros, PilarClarke, BarryPérez-Zepeda, Mario UlisesClay, FelixPerkinson, Margaret A.Cock, CharlesPetretto, Donatella RitaCornell, Charles N.Pizzorni, NicoleCorsonello, AndreaPluta, Michał P.Cravello, LucaPolinder-Bos, HarmkeCronkite, Ruth C.Posluszny, Joseph ACusumano, AnaPowers, BeckyD’Orazi, ValerioPulle, Ranjeev ChrysanthD’Arrigo, SoniaQuintas, JoãoDavis, JenniferRachon, DominikDe Vincentis, AntonioRadoja, IvanDe Wazieres, BenoîtRadzevičienė, AurelijaDell, ColleenRamaswamy, RavishankarDening, TomReider, Lisa M.Di Lorenzo, FrancescoRhein, MathiasDitillo, MichaelRoberts, KenDomagala, AlicjaRodríguez-Hurtado, DianaDuchowny, Kate ARogobete, Alexandru FlorinEdes, Thomas E.Rogus-Pulia, NicoleEgberts, AngeliqueRokach, AmiEnders-Slegers, Marie JoséRoque, FátimaFalkenstein, MichaelRugg, ChristopherFernández-Alcántara, ManuelRussell, RichardFernández-Martínez, EliaSacco, EmilioFigueiredo, Isabel V.Sakai, KotomiFisk, Malcolm J.Santamera, AntonioFlores-Guerrero, Jose L.Santric Milicevic, MilenaForma, LeenaSchmidt, TanjaFrost, RachaelSchofield, PatriciaFukuda, HirotsuguSchoon, JanoschG. Smithard, DavidSchoufour, J.D.Gabutti, GiovanniScorziello, AntonellaGallagher, DavidSebastianelli, ArcangeloGarcía-González, VíctorSerban, CostelaGarner, TomSerra-Rexach, Jose AntonioGeldmacher, DavidSganga, FedericaGeorge, AndersonShankar, Kalpana NarayanGeorge, StaceySilva, Ana RitaGijón-Nogueron, GabrielSilva, SamuelGlusman, GustavoSingh, InderpalGoettel, NicolaiSlater, MargaretGolchin, NegarSłomka, ArturHain, DebraSlooter, ArjenHakeem, Faisal F.Smith, JaredHalvorsen, KjellSmith, TobyHamer, OkkaSouders, Dustin J.Hanser, SuzanneSoutherland, JodiHellström, IngridSpeck, Patricia M.Hendry, AnneStehle, PeterHewitt, JonathanStemplewski, RafałHill, CameronSteves, ClaireHill, TomStewart, AlbertHinrichs, TimoStrogatz, DavidHorn, SebastianSumitani, MasahikoHrdy, JiriSun, YiHuff, AlyssaSzeliga, JacekIrvine, LeslieTanaka, MasaruIsola, GaetanoTanzer, MichaelJackson, Alun C.Temple, NormanJagannatha, RaoThorslund, BirgittaJankowski, Catherine M.Tinnirello, AndreaJiang, XiantaTohara, HarukaJones, Corinne A.Tomkinson, GrantJürisson, MikkToscano, MassimilianoJurkowski, Elaine T.Tsakiridis, IoannisKatarina, WilhelmsonTsotsos, Lia E.Katlic, Mark R.Tumosa, NinaKatsavelis, DimitriosUchida, MarcoKaźmierczak-Siedlecka, KarolinaUnsworth, Carolyn A.Keevil, Victoria L.Vaismoradi, MojtabaKelley-Baker, TaraValenzano, AnnaKesting, MarcoVallesi, AntoninoKhurshid, ZohaibVan As, Arjan BastiaanKim, Beom KyungVan Den Beuken-van Everdingen, Marieke H.J.King, Barbara J.van Hoof, JoostKinner, Bernd J.Venusia, CovelliKlobučar, HrvojeVersnel, HuibKnoefel, FrankVerzak, ŽeljkoKobylecki, ChristopherVignatelli, LucaKojima, TaroVink, AnnemiekeKrzych, LukaszVlakveld, WillemKukielka, ElizabethVon Esenwein, SilkeKunutsor, Setor K.Wang, YiLaHue, SaraWard, Katherine T.Larner, AndrewWarfel, Jaycob D.Léonard, GuillaumeWeeks, Lori E.Lewko, JolantaWieczorek, AnetaLima, Julie C.Wiśniewski, AdamLindemann, UlrichWittmann, MariaLindquist, LeeXiao, XiaLinge, HelenaYamamoto, NorioLiu, FangYiengprugsawan, Vasoontara SbirakosLodha, NehaZaletel, MarjanLogan, PatriciaZeiler, MatthiasLomonaco, TommasoZhang, LinglingLópez-Guerra, DiegoZolezzi, MonicaMadhavan, AarthiZucchelli, Alberto

